# Multiple aseptic cellulitis following continuous subcutaneous injection of foslevodopa-foscarbidopa: Two case reports

**DOI:** 10.1016/j.jdcr.2025.10.076

**Published:** 2025-12-17

**Authors:** Manon Blaise, Anaïs Puppo, Alecu Cosmin, Nathalie Cardot-Leccia, Fanny Rocher, Henri Montaudié, Caroline Giordana, Thierry Passeron

**Affiliations:** a Dermatology Department, Centre Hospitalier Universitaire de Nice, Université Côte d’Azur, Nice, France; b Neurology Department, Centre Hospitalier Universitaire de Nice, Université Côte d’Azur, Nice, France; c Department of Pathology, Centre Hospitalier Universitaire de Nice, Université Côte d’Azur, Nice, France; d Department of Pharmacovigilance, Centre Hospitalier Universitaire de Nice, Université Côte d’Azur, Nice, France; e Centre Méditerranéen de Médecine Moléculaire (C3M), INSERM U1065, Université Côte d’Azur, Nice, France

**Keywords:** aseptic cellulitis, foslevodopa-foscapidoda, Parkinson disease

Foslevodopa-foscarbidopa (FF) continuous subcutaneous infusion is an emerging therapy for advanced Parkinson disease (PD). The FF pump provides continuous subcutaneous infusion of phosphorylated levodopa combined with foscarbidopa, a decarboxylase inhibitor. This method bypasses the digestive system, preventing early metabolism of levodopa, resulting in more stable and sustained drug levels in the bloodstream, which lead to more consistent dopamine availability in the brain and improved control of motor symptoms. While this treatment has demonstrated efficacy, over 90% of patients report adverse effects, with the most common being “injection site erythema,” “nodules,” and “cellulitis.”^1^ The severity, etiology, and management of cellulitis induced by FF remain poorly defined. We present 2 cases of severe cellulitis at the FF injection sites.

A man in his 60s with a history of type 2 diabetes, gastric lymphoma in remission, and malnutrition (body mass index of 15) was diagnosed with advanced PD for 12 years. He developed motor fluctuations despite treatment by rasagiline, pramipexole, and oral fractioned levodopa-carbidopa-entacapone. A subcutaneous FF infusion was initiated. Three days after pump placement, the patient developed an erythematous, indurated, and painful nodule at the cannula insertion site. A second similar reaction occurred when the catheter was repositioned to a hypochondral location (Fig 1, *A*), followed by a third episode when the cannula was moved to the right arm (Fig 1, *B*). No fever or systemic symptoms were observed, and no mechanical trauma had occurred at the affected site area, nor vaccination. Antibiotic therapy with pristinamycin for 10 days was ineffective. Drainage of clear fluid from the site yielded nonpathogenic bacteria. Histopathological examination revealed superficial and deep dermal inflammatory fibrosis extending into the superficial subcutis, with a polymorphous inflammatory infiltrate composed of lymphocytes, eosinophils, and neutrophils organized around adipocytes, without detectable microorganisms, consistent with aseptic cellulitis (Fig 2, *A*-*D*). By day 10 following the appearance of the nodules, treatment with FF was discontinued and the catheter was removed. On the same day, topical therapy with betamethasone dipropionate 0.05% once daily was initiated. Symptoms markedly improved within the first 2 days and resolved completely after 7 days of treatment.

**Fig 1:**
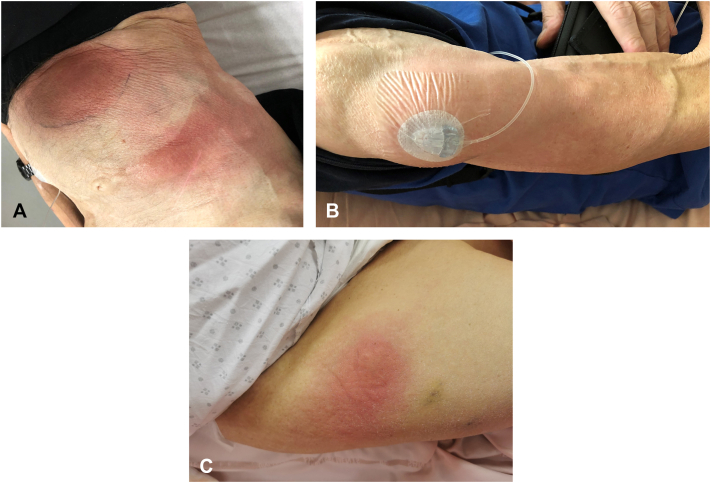
Clinical presentation. **A,** Patient 1: erythematous, indurated, and painful nodule at the cannula insertion site in hypochondral location. **B,** Patient 1: second and similar reaction at the cannula insertion in the right arm location. **C,** Patient 2: erythematous, painful nodule at the cannula insertion site on the right thigh location.

**Fig 2:**
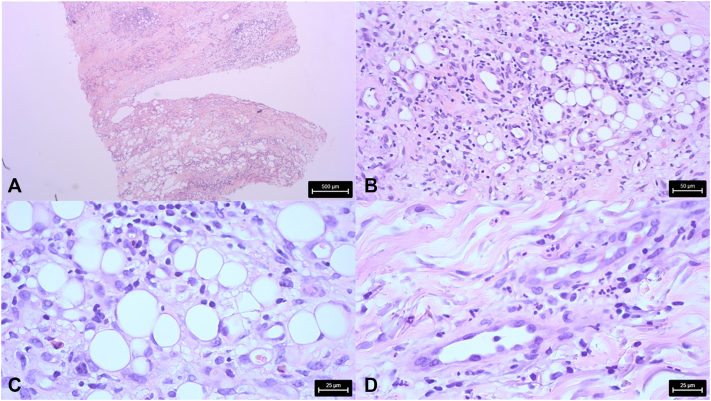
Histological correlation of patient 1. **A,** Superficial and deep dermal fibrosis and infiltrate extending into the superficial subcutis (H&E 25). **B,** Polymorphous inflammatory infiltrate in subcutaneous tissue (H&E 200). **C,** Infiltrate with lymphocytes and some eosinophils around adipocytes (H&E 400). **D,** Interstitial neutrophils (H&E 400).

The second case is a woman in her 70s with a 20-year history of advanced PD, osteoarthritis, and overweight (body mass index 28). She had developed motor fluctuations and disabling dyskinesia. FF was introduced, and 10 days later, the patient developed an erythematous, painful nodule at the cannula insertion site. A similar reaction occurred when the pump was relocated to the right thigh (Fig 1, *C*). No local factors contributed to cellulitis. The patient exhibited an inflammatory syndrome, characterized by fever and elevated C-reactive protein levels, secondary to a flare of shoulder chondrocalcinosis that resolved within 3 days. Ultrasound showed swelling with tissue infiltration but no abscess. A skin biopsy from the right thigh revealed polymorphous inflammatory infiltrate with predominantly neutrophils, with no granulomatous pattern. The lesions improved with potent topical steroids, and FF treatment was resumed without recurrence.

Preclinical studies on FF have highlighted a significant incidence of adverse skin reactions, including injection-site cellulitis.^2^, ^3^, ^4^ Phase 3 clinical trials reported infusion-site cellulitis in 27.7% of patients, typically diagnosed based on clinical signs. Cultures of infusion sites rarely identified bacterial pathogens, and most adverse events were graded as mild to moderate, managed with oral antibiotics.^5^ Distinguishing between injection-site cellulitis and infection cellulitis presents a diagnostic and therapeutic challenge. Infusion-site cellulitis can mimic infectious processes, leading to overlapping signs and symptoms. Furthermore, the diagnosis can be complicated by the fact that certain antibiotics used to treat cellulitis, such as doxycycline, possess both antibacterial and anti-inflammatory effects.^6^ Consequently, improvement following antibiotic treatment does not definitively confirm the presence of cellulitis. No prognostic factors for skin cellulitis during FF treatment have been identified. It is hypothesized that reactive inflammation may occur due to the high concentration of the drug in subcutaneous adipose tissue.

These 2 cases of multiple aseptic cellulitis following FF treatment suggest that the phenomenon may be more severe than previously reported in phase 3 trials. Both cases support the theory of an aseptic reaction, highlighting the efficacy of potent topical steroids as the primary therapeutic approach. While systematic antibiotic therapy should generally be avoided in mild, noninfectious infusion-site reactions, empirical antibiotic therapy may be warranted until the culture report, when infectious cellulitis is suspected, particularly if systemic signs are present or the lesions worsen. Physicians managing PD patients should be aware of this potential adverse event and inform patients accordingly. Additionally, postmarketing surveillance is crucial to monitoring the frequency of severe skin reactions, particularly aseptic cellulitis.

## Conflicts of interest

None disclosed.
